# A novel c. 204 Ile68Met germline variant in exon 2 of the mutL homolog 1 gene in a colorectal cancer patient

**DOI:** 10.3892/ol.2014.2666

**Published:** 2014-11-04

**Authors:** PAVEL VODICKA, FABIAN CAJA, VERONIKA VYMETALKOVA, PAVEL PROCHAZKA, LUDMILA VODICKOVA, LUCIE SCHWARZOVA, JANA SLYSKOVA, RAJIV KUMAR, MICHAELA SCHNEIDEROVA

**Affiliations:** 1 Department of Molecular Biology of Cancer, Institute of Experimental Medicine, Academy of Sciences of the Czech Republic, Prague 14220, Czech Republic; 2 Department of Molecular Genetics, Institute of Biology and Medical Genetics, First Faculty of Medicine, Charles University in Prague, Prague 12800, Czech Republic; 3 Department of Immunology and Gnotobiology, Institute of Microbiology, Academy of Sciences of the Czech Republic, Prague 14220, Czech Republic; 4 Department of Toxicogenomics, National Institute of Public Health, Prague 10042, Czech Republic; 5 Division of Molecular Genetic Epidemiology, German Cancer Research Center, Im Neuenheimer Feld, Heidelberg 69121, Germany; 6 Department of Surgery, General University Hospital in Prague, Prague 12800, Czech Republic

**Keywords:** MutL homolog 1, germline mutation, colorectal cancer

## Abstract

Mutations in the mutL homolog 1 (*MLH1)* gene are frequent in patients with hereditary non-polyposis colorectal cancer (CRC). The MLH1 gene was screened for mutations in patients with sporadic CRC. The nucleotide sequences for all 19 exons of *MLH1* were analyzed by high resolution melting and sequenced in a group of 104 sporadic CRC patients, and the results were verified in a replication group of 1,095 patients and 1,469 controls. Different melting profiles for exon 2 of the *MLH1* gene were observed in the germline DNA of one patient. Sequencing of the patient’s DNA resulted in the identification of a heterozygous C>G variant at c.204, which resulted in an Ile68Met change in the amino acid. A detailed search of the National Center for Biotechnology Information and the 1000 Genomes databases indicated that the detected variant was unique. According to the SIFT and PolyPhen-2 algorithms, the substitution of Ile to Met was predicted to decrease the activity of the MLH1 protein. The newly identified, functional germline variant was not present in any other CRC patient or control. Thus, a novel germline variant in the *MLH1* gene was identified, representing a rare event in sporadic CRC. The occurrence and relevance of this mutation in other types of cancer requires additional investigation.

## Introduction

MutL homolog 1 (*MLH1*), a constituent gene in the mismatch repair pathway, carries germline mutations in individuals with Lynch syndrome, also termed hereditary non-polyposis colorectal cancer (HNPCC). The gene is reported to acquire 300 different germline mutations, which in addition to mutations in other genes involved in mismatch repair pathway, mainly mutS homolog 2 (*MSH2*), predispose individuals to the disease (1). MLH1, a key protein of the mismatch repair process, contains interaction domains for MutS homologs, including MSH2, MSH3 and MSH6, postmeiotic segregation increased 2 (PMS2), MLH3 and PMS1(2). The heterodimers formed by MLH1 recruit proteins for the excision and repair synthesis.

The germline mutations in *MLH1* in HNPCC include nucleotide substitutions, which result in missense, nonsense or splicing errors and also comprise insertions/deletions. A number of founder mutations, which account for a high proportion of mutations in families with HNPCC, which have been reported in patients with Lynch syndrome (3). In addition, certain non-pathogenic mutations in exons and introns of the gene have also been reported (4,5). The *MLH1* gene is highly polymorphic, with >1,600 variants reported to date (http://genecards.org/cgi-bin/carddisp.pl?gene=MLH1&search=mlh1%23snp).

In this study, a novel germline mutation in *MLH1* in a patient with sporadic colorectal cancer (CRC) is reported, which was detected during the whole genome mutational screening. In addition, a total of 1,095 sporadic CRC patients and 1,469 controls were tested for the detected mutation.

## Materials and methods

### Study population

This study included a group of 104 newly diagnosed CRC patients with DNA extracted from their tumor tissues, adjacent healthy mucosa and peripheral blood tissues. A replication group included 1,095 CRC patients and 1,469 controls from whom DNA was extracted from peripheral blood lymphocytes. The information regarding the CRC cases and controls included in the replication group is shown in Table I and has been described previously (6,7). Patients included in this study attended the General University Hospital (Prague, Czech Republic), Thomayer Hospital (Prague, Czech Republic), Central Military Hospital (Prague, Czech Republic), Faculty Hospital (Brno, Czech Republic), Regional Hospital Benesov (Benesov, Czech Republic), Regional Hospital Liberec (Liberec, Czech Republic), Hospital Na Plesi (Nova Ves pod Plesi, Czech Republic), Regional Hospital Pribram (Pribram, Czech Republic), Masaryk Regional Hospital (Ústí nad Labem, Czech Republic), Tomas Bata Regional Hospital (Zlín, Czech Republic) or Jihlava Regional Hospital (Jihlava, Czech Republic). This study was approved by the ethics commitee of the General University Hospital in Prague (Prague, Czech Republic) and written informed consent was obtained from all patients.

### Mutation screening in CRC patients

DNA from tumor tissues, healthy mucosa and blood tissues was extracted using QIAamp DNA Mini Kit and QIAcube (Qiagen, Hilden, Germany). The extracted DNA of the 104 newly diagnosed CRC patients were subjected to mutation detection by high-resolution melting (HRM) using LightCycler® and a 480 High Resolution Melting Master® kit (Roche Diagnostics GmbH, Mannheim, Germany). Polymerase chain reaction amplicons were designed to scan the *MLH1* gene using HRM analysis. All 19 exons in the *MLH1* gene were screened. The region containing exon 2 was amplified using primers with the following sequence: Forward, 5′-AGTTTGTTATCATTGCTTGGCTCAT-3′ and reverse, 5′-TCCAGAACAGAGAAAGGTCCTGACT-3′ (8). The 10-μl reaction mixture contained 20 ng genomic DNA, 0.4 mM of each primer and 3 mM MgCl_2_. The reaction conditions were as follows: Activation step at 95°C for 10 min followed by 45 cycles of 95°C for 15 sec, 60°C for 15 sec, 72°C for 25 sec and 72°C for 7 min. Samples with positive HRM signals were further analyzed by sequencing. Sequencing reactions were carried out in a total volume of 10 μl containing 2 μl Big Dye^®^ Terminator v1.1 Cycle Sequencing kit (Applied Biosystems, Life Technologies, Foster City, CA, USA), 2.4 μl H_2_O, 0.3 μl DNA template and 0.3 μl of one primer. The reaction products were purified by ethanol precipitation and sequenced by automated sequencing (Applied Biosystems^®^ 3130 Genetic Analyzer; Applied Biosystems, Life Technologies). The reference sequence of the *MLH1* gene (NG_007109.1) was obtained from the National Center for Biotechnology Information database (http://www.ncbi.nlm.nih.gov/nuccore/NG_007109.1?from=4863&to=62359&report=genbank).

### Genotyping of the replication group

DNA samples from the replication group were genotyped for the novel germ-line variant. The DNA samples from CRC patients and controls were analyzed at the K Bio Science facility (K Bio Science UK Ltd., Hoddesdon, UK) (protocol available at http://www.kbioscience.co.uk/reagents/KASP_manual.pdf) under conditions described previously (6).

## Results

**Figure 1:**
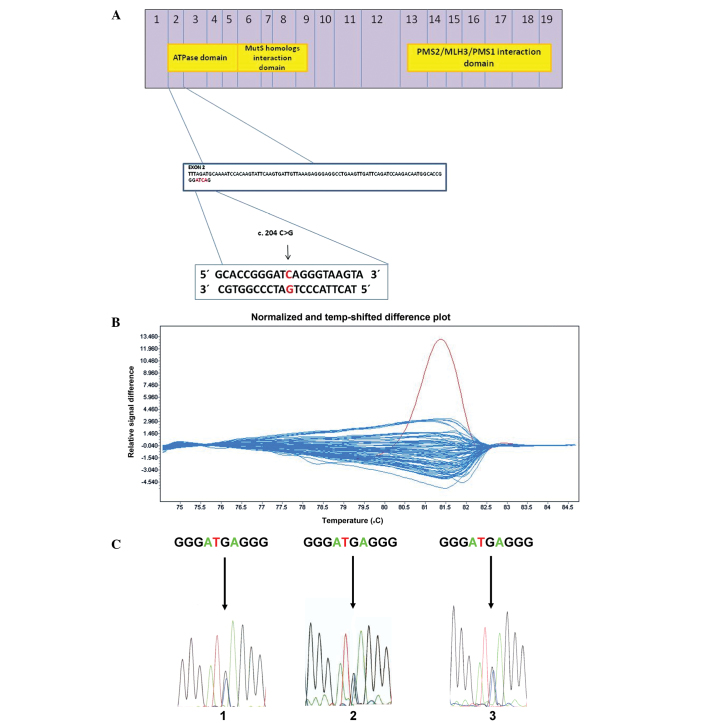
HRM analysis and DNA sequencing of exon 2 of the *MLH1* gene. (A) Diagram of the MLH1 protein in scale. Numbers inside the blue boxes indicate the numbers of exons from which each part of the protein is translated. The three yellow boxes inside represent the ATPase domain, the MutS homolog interaction domain and the PMS2/MLH3/PMS1 interaction domain. A new germ-line variant in exon 2 is located at position c. 204 C>G, p. Ile68Met, corresponding to the gene region coding a part of ATPase domain of MLH1 protein. The position c. 204 is shown by the black arrow. (B) HRM analysis of exon 2 in tumor DNA samples. The difference plot chart shows relativity between DNA melting temperature and relative intensity of emitted fluorescence. Each curve represents the melting of one DNA sample. The DNA sample bearing the novel gene variant exhibits a different melting profile, which is highlighted by the red color in comparison with the blue-colored melting profiles of wild-type DNA samples. This discrepancy of different melting curves is caused by nucleotide change in the analyzed DNA sequence. The red curve represents the melting of the DNA sample which bears the newly identified variant, c. 204 C>G, p. Ile68Met, in tumor tissue. To verify the possible germ-line origin of the variant, HRM was performed in healthy tissue and peripheral blood DNA samples. Both samples were positive for the same variant (data not shown). (C) Comparison of DNA sequencing plots of three different DNA samples obtained from the same patient bearing new heterozygous germ-line gene variant c. 204 C>G, p. Ile68Met. 1, tumor DNA sample; 2, healthy mucosa tissue DNA sample; and 3, peripheral blood DNA sample. MLH1, mutL homolog 1; PMS2, postmeiotic segregation increased 2;HRM, high-resolution melting.

In the initial group, 1/104 sporadic CRC patients exhibited a single nucleotide variant at codon 204 within exon 2 of *MLH1* in tumor tissue and mucosa, as well as in blood lymphocytes DNA. A change of the base C to G resulted in Ile68Met change in the amino acid residue (Fig. 1). The carrier of the newly identified variant was a 59 year old female with rectal cancer (T3N0M0 stage). Although the family history of the patient was unavailable, the patient had been treated for schizophrenia for 15 years. The tumor was microsatellite-stable, the CpG sites on the *MLH1* promoter were not methylated and the expression of MLH1 protein was not altered in the tumor, when compared with the adjacent mucosa (data not shown). Genotyping of the replication revealed that there were no carriers of the newly identified variant Ile68Met in the CRC or control groups.

## Discussion

In this study, a unique variant c. 204 C>G, p. Ile68Met in exon 2 of the *MLH1* gene was identified in a patient with sporadic CRC. The mutation was germline and was also detectable in the DNA of tumor tissue, colon mucosal tissue and DNA of the peripheral lymphocytes of the patient. Predictive algorithms SIFT (9) and PolyPhen-2 (http://genetics.bwh.harvard.edu/pph2/) indicated that the amino acid change from isoleucin to methionin may influence the functionality of the MLH1 protein. A 3D model of the MLH1 protein demonstrated that amino acid residue 68 isoleucin is located within the enzymatic core that interacts with ATP molecules (http://www.ncbi.nlm.nih.gov/Structure/mmdb/mmdbsrv.cgi?uid=90223). Therefore, a substitution by methionine may decrease MLH1 activity. Similarly, a variant in an HNPCC patient at the position c.203 T>A, p. Ile68Asn was previously shown to be deleterious (10). Furthermore, a homologous substitution in yeast was found to cause loss of function in a mismatch repair assay (11).

The identification of a novel germline variant in *MLH1* with putative impact on the protein function is of significant importance in CRC. *MLH1* is the most frequently mutated gene in HNPCC and is also often altered in sporadic forms (12). The novel variant in *MLH1* gene presents a rare event in sporadic CRC, which was identified in 1/1,199 patients. The effect of the mutation, whether a causal germline variant or a rare polymorphism, remains to be determined. Due to the patient history, an association between the novel mutation and mental illness must not be excluded. Neurodegenerative diseases, such as Huntington’s disease, are also known to exhibit alterations in mismatch repair genes (13).

In conclusion, in the present study a novel *MLH1* mutation was detected in a patient with sporadic CRC. The functionality of the novel c. 204 C>G, p.Ile68Met variant in exon 2 of *MLH1* gene remains to be determined experimentally, along with its occurrence and relevance in other cancer types.

## Figures and Tables

**Table I:** Characteristics of the study population.

Characteristic	CRC cases (n=1095)	Control group I, CFCC (n=688)	Control group II, HBDV (n=781)	All controls (n=1469)	OR	95% CI	P-value^a^
Tumor localization							
Colon	725	-	-	-			
Rectum	370	-	-	-			
Age (years)							
47≤	94	164	427	591	Ref.		
48–55	208	145	277	422	3.10	2.36–4.09	≤0.01
56–65	370	209	77	286	8.13	6.25–10.66	≤0.01
>65	423	170	0	170	15.37	11.66–20.44	≤0.01
Gender							
Female	435	317	343	660	Ref.		
Male	660	371	438	809	1.23	1.05–1.45	0.01
BMI							
23.7≤	184	154	215	369	Ref.		
23.7–26.2	192	147	213	360	1.07	0.83–1.37	0.61
26.3–28.9	226	139	184	323	1.40	1.10–1.79	0.01
>28.9	222	172	157	329	1.35	1.06–1.73	0.02
Smoking history							
No	536	364	451	815	Ref.		
Yes^b^	501	254	327	581	1.31	1.12–1.54	≤0.01
Family history of CRC							
No	726	486	718	1204	Ref.		
Yes	144	90	52	142	1.68	1.31–2.16	≤0.01
Address							
City	511	338	614	952	Ref.		
Suburbs	128	118	53	171	1.39	1.08–1.79	0.01
Countryside	242	157	112	270	1.67	1.36–2.05	≤0.01
Education							
Basic	266	171	53	224	Ref.		
Medium	469	327	492	820	0.48	0.39–0.59	≤0.01
High	138	114	231	345	0.34	0.26–0.44	≤0.01

a P-value for comparison of CRC cases and all controls.

b Ex-smokers are included into this group.

Basic education, completion of eight years of education at primary and secondary school; medium education, completion of 12 years of education at higher secondary school; high education, completion of 17 years of education at university. In cases where categories do not equal 100%, this is due to missing data. CRC, colorectal cancer; CFCC, cancer-free colonoscopy inspected controls; HBDV, healthy blood donor volunteers; OR, odds ratio, CI, confidence interval; BMI, body mass index.
